# Improved Short-Circuit Current and Fill Factor in PM6:Y6 Organic Solar Cells through D18-Cl Doping

**DOI:** 10.3390/nano13212899

**Published:** 2023-11-03

**Authors:** Jianjun Yang, Xiansheng Wang, Xiaobao Yu, Jiaxuan Liu, Zhi Zhang, Jian Zhong, Junsheng Yu

**Affiliations:** 1 College of Electron and Information, University of Electronic Science and Technology of China, Zhongshan Institute, Zhongshan 528402, China; 2 School of Optoelectronic Science and Engineering, University of Electronic Science and Technology of China, Chengdu 610054, Chinajsyu@uestc.edu.cn (J.Y.)

**Keywords:** ternary blend organic solar cell, D18-Cl, double-donor type, short-circuit current

## Abstract

Based on the PM6:Y6 binary system, a novel non-fullerene acceptor material, D18-Cl, was doped into the PM6:Y6 blend to fabricate the active layer. The effects of different doping ratios of D18-Cl on organic solar cells were investigated. The best-performing organic solar cell was achieved when the doping ratio of D18-Cl reached 20 wt%. It exhibited a short-circuit current of 28.13 mA/cm^2^, a fill factor of 70.25%, an open-circuit voltage (*Voc*) of 0.81 V, and a power conversion efficiency of 16.08%. The introduction of an appropriate amount of D18-Cl expanded the absorption spectrum of the active layer, improved the morphology of the active layer, reduced large molecular aggregation and defects, minimized bimolecular recombination, and optimized the collection efficiency of charge carriers. These results indicate the critical importance of selecting an appropriate third component in binary systems and optimizing the doping ratio to enhance the performance of ternary organic solar cells.

## 1. Introduction

The advantages of organic solar cells, such as their lightweight nature, simple fabrication processes, and the ability to be made into flexible large-area devices, have attracted the interest of researchers [1,2,3,4]. Currently, the commonly used binary system in organic solar cells has a limited native heterojunction structure due to the active layer material’s intrinsic absorption spectral range. This limitation hinders the cell’s ability to absorb a wider spectrum of photons, ultimately restricting the enhancement of photovoltaic conversion efficiency. To address this challenge, researchers have proposed two alternative structures for organic solar cells: tandem stacked solar cells and ternary system solar cells. Tandem-stacked solar cells involve the sequential arrangement of sub-cells with distinct absorption spectra, effectively broadening the overall absorption range [5,6,7]. However, the complex fabrication process associated with tandem-stacked solar cells hinders their practical production applications. On the other hand, ternary system organic solar cells involve the introduction of a third component, such as organic small molecules or polymers, through doping into the binary system [8,9,10,11]. This approach allows for the preparation of a hybrid active layer that overcomes the complexities of the tandem stacked structure while simultaneously broadening the absorption spectral range [12,13,14,15]. Furthermore, the use of different dopant materials with varying intrinsic properties enables the modification of the active layer’s morphology, enhances exciton utilization, and improves carrier mobility [16,17,18,19,20,21,22]. 

The concept of the ternary system was initially introduced by M.C. Quiles et al., who incorporated a third material component into the binary system of the P3HT:PCBM hybrid active layer to develop ternary organic solar cells [23]. Kung-Hwa et al. demonstrated the use of ternary blends, featuring a high-band-gap small molecule (SM-4OMe), a low-band-gap polymer (PTB7-TH), and a fullerene, as active layers in single-junction organic photovoltaic devices, resulting in a power conversion efficiency of 10.4% [24]. In a study by Zhao et al., PBDB-T fluorination was combined with a newly synthesized IT-4F receptor, resulting in a device that achieved a record-breaking power conversion efficiency (PCE) of 13.1% [25]. Huifeng Yao’s group further enhanced the performance by doping PTO2 as the third element in the PBDB-TF:IT-4F system, leading to an increased photovoltaic conversion efficiency of 15.6% [26]. Yu-Che Lin et al. presented a novel terpolymer acceptor for all-polymer organic photovoltaics, achieving a high power conversion efficiency of 16.7% in single-junction solar cells [27]. Ming-Ao Pan et al. achieved organic solar cells with over 17% efficiency by incorporating PC71BM into the PBDB-T-2F:Y6 system [28]. Similarly, Yunhao Cai et al. incorporated a novel non-fullerene acceptor (NFA), L8-BO-F, into a PM6:BTP-eC9 mixture, optimized the morphology of the ternary blends, and successfully fabricated organic solar cells with a conversion efficiency of 18.2% [29]. Non-fullerene acceptor materials have emerged as promising organic materials in the field of organic solar cells due to their excellent optical absorption properties and tunable energy levels [30,31,32,33,34,35]. Although the incorporation of a third material component has shown promise in boosting the PCE, the achieved *Jsc* values in reported studies remain below the desired levels [36,37,38]. It is crucial to explore novel strategies to optimize the ternary blend composition and morphology, as well as the charge transport pathways, to overcome the challenges associated with limited *Jsc*. The introduction of the PM6:Y6 system has paved the way for innovative research directions. Karki et al. [39] were the first to explore the structure-performance relationship of this high-performance BHJ (bulk heterojunction) blend and employed techniques such as photodirected atomic force microscopy, grazing-incidence wide-angle X-ray scattering, and solid-state 19F magic angle spinning nuclear magnetic resonance spectroscopy to analyze phase separation and molecular interactions within the thin film. These efforts resulted in a remarkable efficiency of 15%. Furthermore, Wang et al. [40] conducted a study on the PM6:Y6 blend using broadband transient absorption (TA) spectroscopy. Their research elucidated that the hole transfer pathway for photogenerated charges in the blend is mediated by internal excitations. As a result, they achieved an impressive efficiency of 17% in a single-junction organic solar cell. Simultaneously, high photovoltaic performance exceeding 18% was achieved using the PM6:Y6 binary blend system [41]. By introducing polymer donors, small molecule donors, fullerene acceptors, and small molecule acceptors into the PM6:Y6 binary system, improvements in surface morphology, optimized phase separation, enhanced solubility, and reduced energy losses were realized, consequently elevating photon harvesting capabilities and facilitating exciton dissociation and charge transport.

This paper investigates the effects of doping a novel non-fullerene material, D18-Cl, into the PM6:Y6 binary system and its impact on the performance of organic solar cells. Different doping ratios of D18-Cl are examined. The analysis of metallographic and AFM diagrams reveals several improvements in the organic solar cell resulting from the addition of D18-Cl. Further research has also confirmed the positive effect of D18-Cl on improving short-circuit current density. Ultimately, the dual-donor organic solar cell achieved an enhanced short-circuit current (*Jsc*) of 28.13 mA/cm^2^.

## 2. Materials and Methods

### 2.1. Materials

In the present study, the materials were sourced as follows: PM6 and Y6 were purchased from Solarmer Materials Inc. (Beijing, China); D18-Cl, zinc acetate dihydrate, molybdenum trioxide, and chloroform were obtained from Nanjing Zhiyan Technology Co., Ltd. (Nanjing, China); ethylene glycol monomethyl ether and ethanolamine were procured from Maclin Biochemical Technology Co., Ltd. (Shanghai, China); silver materials were acquired from Zhongnuo New Materials (Beijing) Technology Co., Ltd. (Beijing, China); acetone was sourced from Beijing Chemical Reagent Company (Beijing, China); isopropanol was obtained from Sigma-Aldrich (Burlington, MA, USA); and ITO transparent conductive glass was purchased from Nuozhuo Technology Co., Ltd. (Wuhan, China).

### 2.2. ITO Glass Substrate Treatment

ITO (indium tin oxide) transparent conductive glass substrates were placed on substrate holders. Subsequently, a sequential ultrasonic cleaning process was performed using deionized water, acetone, and isopropanol solutions. The ultrasonic cleaning was conducted at a frequency of 90 Hz, maintaining a temperature of 40 °C, with a cleaning duration of 20 min. After the cleaning process, the glass substrates were placed in an oven for drying. Following that, a 20-min UV irradiation was applied to the substrates for ultraviolet cleaning.

### 2.3. Electron Transport Layer Fabrication

For the fabrication of the electron transport layer, ethylene glycol monomethyl ether served as the solvent, zinc acetate acted as the solute, and ethanolamine was introduced as a catalyst to facilitate the formation of ZnO. The solute-to-solvent mass ratio was set at 109 mg/mL, with an additive-to-solvent ratio of 29 μL/mL. The pre-weighed zinc acetate was transferred to a reagent bottle, and, with the use of a pipette, ethylene glycol, monomethyl ether, and ethanolamine were gradually added. Subsequently, the solution was sealed upon the introduction of a magnetic stir bar and placed on a magnetic stirrer. Stirring was conducted at a temperature of 25 °C and a rotational speed of 550 rpm for a minimum of 5 h. The solution was then allowed to equilibrate for approximately 8 h in a refrigerator to ensure complete dissolution of the ZnO precursor. Subsequently, the ZnO solution was filtered through a disposable microporous membrane with a pore size of 0.22 μm using a syringe. The prepared substrates were mounted onto a spin coater, and 40 μL of the ZnO solution was dispensed onto each substrate at a spin-coating speed of 5000 rpm. The deposited films were annealed at 200 °C for 1 h to attain stable transport layer-thin films.

### 2.4. Photoactive Layer Preparation

The preparation of the photoactive layer involved the measurement of PM6, D18-Cl, and Y6 in various doping ratios, specifically 1:0:1.2, 0.9:0.1:1.2, 0.8:0.2:1.2, and 0.7:0.3:1.2. The organic photoactive layer materials were weighed and placed into brown sample bottles. Chloroform was employed as the solvent, with a specific solute-solvent concentration ratio of 13 mg/mL. Afterward, a magnetic stirrer was introduced into the sample bottles, and the caps were securely fastened with 3M tape to prevent chloroform evaporation and maintain the solution concentration. The samples were placed on a magnetic stirrer with stirring parameters set at 600 rpm and a temperature of 30 °C for dissolution over a period of 8 h. Prior to use, the dissolution status of the photoactive layer materials was examined. If complete dissolution was not achieved, the stirring speed was increased to 900 rpm, and the temperature was elevated to 45 °C, ensuring thorough solution dissolution. Afterward, the ZnO thin film substrates that had been previously annealed were placed on a spin coater, and the spin-coating parameters were set to 3800 rpm for 40 s. Using an adjustable pipette, 20 μL of the solution was dispensed onto each substrate to rapidly form a film. Upon completion of spin coating, the substrates were promptly transferred to a hotplate set at 105 °C for annealing, with a duration of 10 min.

### 2.5. Hole Transport Layer and Metal Anode Preparation

The hole transport layer and metal anode were prepared using a high-vacuum thermal evaporation apparatus. Initially, the substrates were embedded in the evaporation mask and firmly secured using high-temperature adhesive tape to ensure close contact between the mask and substrate, preventing an increase in device area and fuzzy edges that could affect experimental accuracy. Subsequently, the evaporation chamber door was opened, and the requisite amounts of MoO_3_ powder and Ag particles were placed into their respective evaporating boats. Evaporation of MoO_3_ commenced once the vacuum gauge reading reached 8 × 10^−4^ Pa. The evaporation rate was manually adjusted to 0.2 Å/s and stabilized at this rate before opening the evaporation boat and sample mask. MoO_3_ deposition concluded when the thickness monitor recorded a film thickness of 15 nm. After a brief cooling period, Ag electrode deposition was carried out at an evaporation rate of 2 Å/s, ultimately resulting in a final thickness of 150 nm. Following the completion of evaporation, the fabricated ternary organic solar cell devices were removed after adequate cooling.

## 3. Results and Discussion

**Figure 1:**
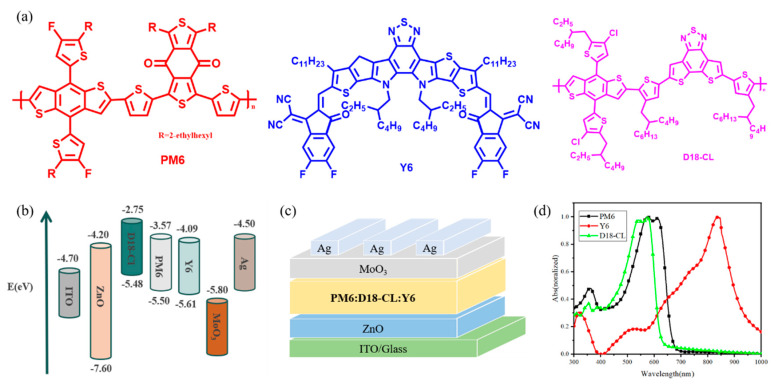
(**a**) The chemical structures of PM6, Y6, and D18-Cl. (**b**) Energy level alignments of the various functional layers. (**c**) The organic solar cell device structure. (**d**) Normalized absorption spectra of active layer blend materials.

Figure 1a,b depicts the chemical structures of PM6, Y6, and D18-Cl, along with the energy level ordering of each functional layer. As shown in Figure 1c, the inverted devices were fabricated using the glass/indium tin oxide (ITO), zinc oxide/active layer/MoO_3_/Ag structure. The selection of new materials primarily considers the peak light absorption range. Specifically, D18-CL exhibits peak light absorption between 500 nm and 600 nm, PM6 between 550 nm and 650 nm, and Y6 between 750 nm and 850 nm. As shown in Figure 1d, the incorporation of the third metamaterial, D18-CL, broadens the absorption of the active layer in the blue visible range, thereby enhancing the overall absorption spectrum coverage of the device. Additionally, the LUMO energy levels of D18-Cl, PM6, and Y6 are −2.75 eV, −3.75 eV, and −4.09 eV, respectively, while the corresponding HOMO energy levels are −5.48 eV, −5.50 eV, and −5.61 eV. This arrangement forms a trapezoidal structure that enhances the transport efficiency of free electrons and holes following exciton dissociation. Notably, D18-Cl possesses a higher LUMO energy level than PM6, creating a stronger built-in electric field at the device heterojunction, thereby facilitating a reduction in electron-hole recombination.

**Figure 2:**
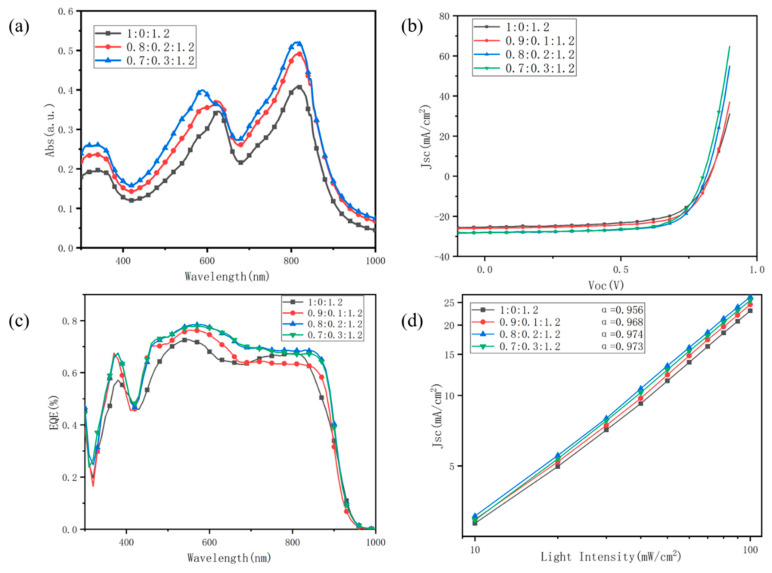
(**a**) Absorption spectra of active layer films with different doping ratios. (**b**) J-V curves of D18-Cl with different doping blending ratios. (**c**) EQE of D18-Cl with different doping blending ratios. (**d**) Light intensity dependence plot of *Jsc*.

Experimentally, it was observed that the addition of a third-element material had a favorable impact on the device. However, increasing the amount of D18-Cl did not necessarily result in better device performance. Based on the PM6:Y6 binary system, there exists an optimal doping ratio of D18-Cl to PM6 that yields the best overall device performance. In this study, organic solar cells were prepared with different doping mass ratios of D18-Cl:PM6:Y6, specifically 0:1:1.2, 0.1:0.9:1.2, 0.2:0.8:1.2, and 0.3:0.7:1.2. To investigate the impact of increasing the doping ratio on the absorption properties of the active layer films, absorption spectra were measured using a UV-visible near-infrared spectrophotometer. As shown in Figure 2a, it can be observed that as the doping ratio increases, the device’s absorption is enhanced in the 400 nm–600 nm spectral range. Additionally, the absorption peak undergoes a leftward shift and stabilizes at 600 nm. This suggests that the introduction of D18-Cl improved absorption across the visible spectral range, thereby broadening the absorption spectrum. Moreover, there is an increase in absorbance between 300 nm and 400 nm, indicating that D18-Cl also promotes enhanced UV absorption. Furthermore, the peak at 800 nm is elevated, indicating improved absorption in the near-infrared region as a result of introducing D18-Cl.

Subsequently, the J-V curves of inverted bulk heterojunction-type devices with different doping co-mingling ratios were measured using the OAI simulator system. The performance parameters corresponding to four doping ratios of PM6:D18-Cl:Y6, namely 1:0:1.2, 0.9:0.1:1.2, 0.8:0.2:1.2, and 0.7:0.3:1.2, are presented in Figure 2b. It can be observed that, under the condition of a constant total donor-acceptor ratio of 1:1.2, the photovoltaic performance of the cell devices initially increases and then decreases with an increasing doping ratio. Table 1 provides the key performance parameters at different doping ratios. The calculation of the fill factor and other values can be found in the supplementary material. All data values were indeed the result of averaging over multiple experiments. For each set of doping ratio experiments, we prepared thirty devices, and the average values were obtained from the best-performing fifteen devices. It can be seen from the table that the open-circuit voltage remains in the range of 0.8–0.83 V, indicating that the increase in the doping ratio of D18-Cl has minimal effect on the open-circuit voltage. However, there is a notable enhancement in short-circuit current density, which follows a certain regularity. Specifically, the short-circuit current density increases from 25.36 mA/cm^2^ to 28.13 mA/cm^2^ and then slightly decreases to 28.01 mA/cm^2^. This can be primarily attributed to the introduction of the D18-Cl donor, which improves exciton dissociation efficiency and enhances the mobility of free electrons and holes. The filling factor shows an increasing trend followed by a decrease with increasing D18-Cl doping percentage, starting at 64.62% and reaching a maximum of 70.2% before decreasing to 69.55%. The filling factor is closely related to the film morphology, and the presence of D18-Cl facilitates the formation of a better vertical phase separation structure in the active layer film, leading to improved exciton dissociation and enhanced collection efficiency of carriers by the cathode. 

Overall, the performance of the binary PM6:Y6 device with undoped D18-Cl material is lower than that of other devices with different D18-Cl doping ratios. Increasing the doping ratio of the D18-Cl material results in varying degrees of enhancement in short-circuit current, fill factor, and photovoltaic conversion efficiency. The highest photovoltaic conversion efficiency of the organic solar cell was achieved at a PM6:D18-Cl doping ratio of 0.8:0.2, corresponding to a 20 wt% D18-Cl doping level. The efficiency increased from 13.52% to 16.08%, representing a 19% improvement compared to the binary PM6:Y6 solar cell. The open-circuit voltage slightly decreased, while the short-circuit current density reached 28.13 mA/cm^2^, and the fill factor was 70.25%. However, further increasing the doping ratio of the D18-Cl material resulted in a decrease in the performance of the organic solar cells. The photovoltaic conversion efficiency decreased by 0.46%, the open-circuit voltage decreased to 0.80 V, and the short-circuit current density decreased to 28.01 mA/cm^2^, with a reduction of only 0.12 mA/cm^2^. Additionally, the filling factor decreased to 69.55%. This situation can be attributed to excessive doping of the D18-Cl material, affecting the co-mingling degree of the solution. This led to increased surface roughness of the spin-coated film, decreased film quality, and increased defects in the interpenetrating lattice structure, thereby affecting charge transport.

According to the formula for calculating short-circuit current density (*Jsc*), the magnitude of *Jsc* is directly proportional to the external quantum efficiency (EQE). The EQE of the organic solar cells at different D18-Cl doping ratios was measured and is shown in Figure 2c. It can be observed that as the D18-Cl doping ratio increases, the EQE values in the 500–700 nm spectral range significantly improve. However, further increasing the D18-Cl doping ratio does not result in a further increase in EQE; instead, there is a slight decrease, which aligns with the trend observed in the variation of short-circuit current density. This further supports the notion that an appropriate D18-Cl doping ratio can expand the absorption spectrum of the film, enhance photon absorption capability, and increase the photocurrent of the solar cell.

Further analysis was conducted to investigate the impact of D18-Cl doping on charge recombination in organic solar cells. The incident light intensity varied from 0 to AM1.5G, and the open-circuit voltage and short-circuit current density were measured to explore their relationship. This analysis aimed to examine the charge recombination in ternary organic solar cell devices with different D18-Cl doping ratios. The relationship between the short-circuit current density (*Jsc*) of the solar cell devices and the incident light intensity can be described by the following equation [42]:

(1) $$J_{sc}\propto P_{\mathrm{light}}^{\alpha }$$

where α is the molecular recombination index, and *P* is the incident light intensity. The equation indicates a power-function relationship between the short-circuit current density and the incident light intensity. A value closer to 1 suggests less recombination of free electrons, which is favorable for efficient charge collection by the anode and cathode. However, it is important to note that some level of molecular recombination is inevitable, and the goal is to minimize it as much as possible to improve the photovoltaic performance. By taking the logarithm of the short-circuit current density and incident light intensity and processing them using software, we obtained the “α“ characteristic curve and corresponding values. Figure 2d shows the values of the molecular recombination index for different PM6:D18-Cl:Y6 ratios. It can be observed that the introduction of D18-Cl as the third component increases the value of “α“, indicating a certain suppression of molecular recombination and improved efficiency of charge collection. However, when the D18-Cl ratio further increases, the value of “α“ decreases. This is mainly due to the increased presence of organic material clusters in the active layer, leading to an increase in the surface roughness of the film and exacerbating molecular recombination.

**Figure 3:**
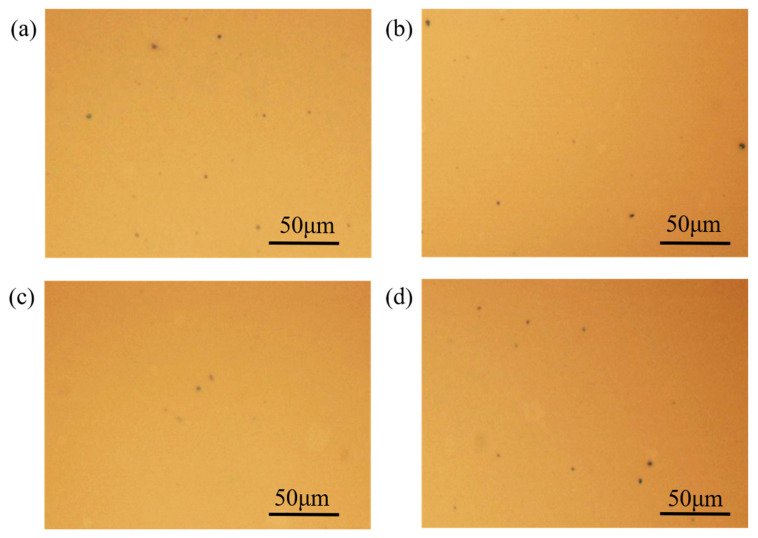
A 100× metallographic micrograph. (**a**) 0 wt%. (**b**) 10 wt%. (**c**) 20 wt%. (**d**) 30 wt%.

Based on the previous analysis, it was found that introducing an appropriate amount of D18-Cl material can improve the short-circuit current density (*Jsc*) and fill factor (FF) of the device. The quality of the active layer film morphology is closely related to Jsc and FF. Therefore, the film surface was characterized using optical microscopy to investigate the presence of large organic material clusters. Figure 3 presents the optical micrographs of the films with D18-Cl doping ratios ranging from 0 wt% to 30 wt%, magnified 100 times. In the case of no D18-Cl doping, the micrograph shows a large number of clusters of donor-acceptor materials on the film surface, with some clusters having a relatively large size. At a doping ratio of 10 wt%, the molecular aggregation on the film surface is reduced, resulting in a decrease in the number and size of the aggregated clusters compared to the undoped film. At a doping ratio of 20 wt%, it can be clearly observed that the large molecular clusters on the film surface are further reduced, indicating an improvement in the overall film quality of the active layer. The reduction in molecular clusters was beneficial for enhancing exciton dissociation efficiency and improving charge carrier mobility, thereby effectively increasing the fill factor. However, when the doping ratio was further increased to 30 wt%, an increase in cluster formation was observed, indicating that excessive D18-Cl leads to molecular aggregation during the film growth process, resulting in a less smooth film. These large molecular clusters were detrimental to exciton dissociation and hindered the formation of good ohmic contacts at the interfaces, thereby affecting charge carrier transport and collection.

**Figure 4:**
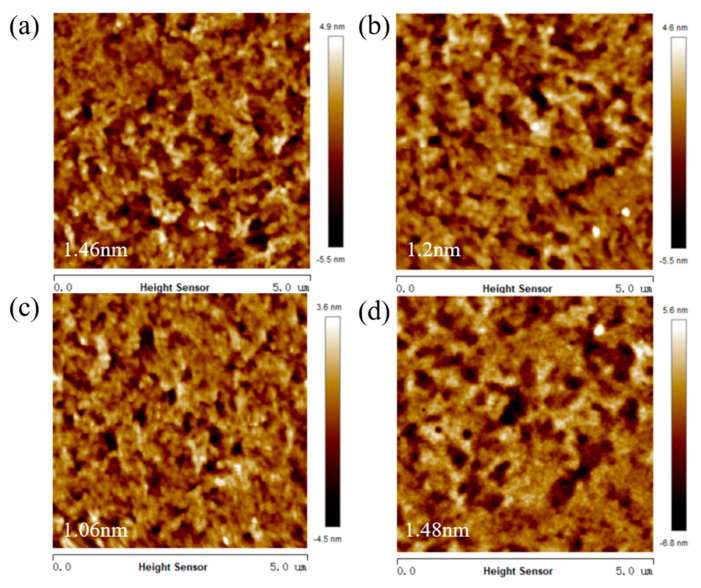
Two-dimensional AFM plots of active layer films with different doping ratios. (**a**) 0 wt%. (**b**) 10 wt%. (**c**) 20 wt%. (**d**) 30 wt%.

**Figure 5:**
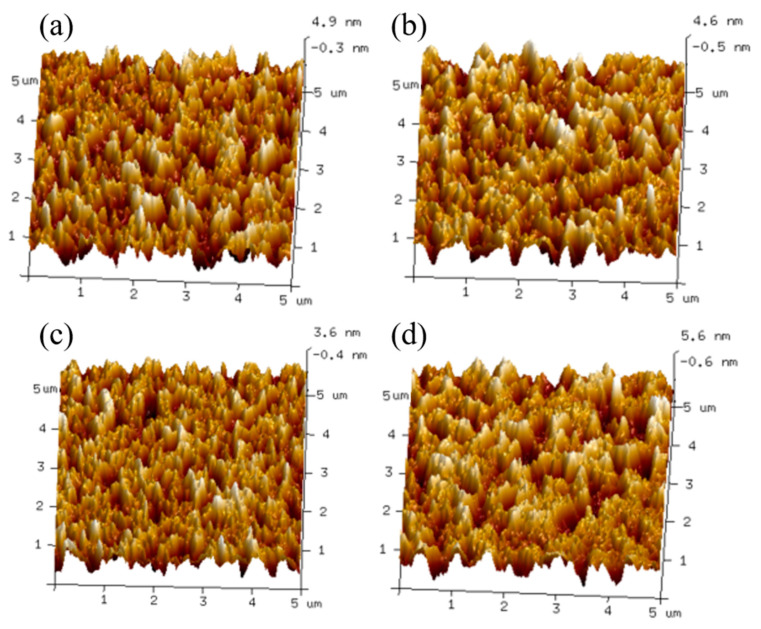
Three-dimensional AFM plots of active layer films with different doping ratios. (**a**) 0 wt%. (**b**) 10 wt%. (**c**) 20 wt%. (**d**) 30 wt%.

In order to obtain more detailed surface morphology characteristics of the active layer film, atomic force microscopy (AFM) was used to test the film morphology. Figure 4 and Figure 5 show the two-dimensional AFM height maps and three-dimensional AFM height maps of the active layer films at different doping ratios of D18-Cl. By analyzing the images, the roughness values of the films within a 5 μm × 5 μm scanning area were obtained. In Figure 4a, which represents the binary heterojunction device without D18-Cl, the surface morphology roughness, as indicated by the Rq value, is 1.46 nm. Figure 4b,c demonstrates that as the D18-Cl doping ratio increases to 20 wt%, the surface of the two-dimensional AFM image becomes smoother, forming a well-interpenetrating nanogrid structure. The Rq value decreases from 1.46 nm to 1.20 nm and further to 0.96 nm. 

In the three-dimensional AFM height map, as shown in Figure 5, the height differences on the film surface, the aggregation of molecules, and the presence of defects are visually displayed. The darkest black points and the lightest white points represent the lowest and highest points on the film surface, respectively. With an increase in the D18-Cl doping ratio, both the black and white areas decreased to varying degrees, indicating a significant reduction in film defects and the aggregation of organic molecules. This suggests that the film had formed an appropriate vertical phase separation structure, which was favorable for the formation of more donor-acceptor interfaces. It can reduce the transmission distance and lower the capability barrier after exciton dissociation, which contributes to the enhancement of device fill factor and short-circuit current density.

However, when the D18-Cl doping ratio further increased to 30 wt%, the Rq value increased to 1.48 nm. The three-dimensional AFM image revealed an increased height difference in the film, indicating intensified aggregation of organic materials. This results in a decrease in the efficiency of exciton dissociation and an exacerbation of double-molecular recombination, leading to a decrease in open-circuit voltage and filling factor, negatively affecting the photovoltaic performance of the device.

## 4. Conclusions

D18-Cl donor material was introduced into the binary system of PM6:Y6 to fabricate double-donor type (D-D-A) ternary heterojunction organic solar cells. The photovoltaic performance of the devices was studied under different doping ratios of D18-Cl. Firstly, through the analysis of the absorption spectra, it was found that the introduction of D18-Cl donor material increased the absorbance of the active layer film, improving the intensity in the range of 500 nm–650 nm. This confirmed the correctness of the material selection strategy. Secondly, experiments have revealed that the addition of an appropriate proportion of D18-Cl effectively reduces large molecular aggregation and defects within the active layer, leading to the formation of a superior vertical structure. These experimental findings validate the ability of D18-Cl to optimize the collection efficiency of charge carriers, thereby confirming its role in promoting an increase in short-circuit current density. Finally, the best overall performance of the organic solar cell was achieved when the mass fraction of D18-Cl was 20 wt%. It exhibited a power conversion efficiency of 16.08%, which was an 18.9% improvement compared to the binary PM6:Y6 organic solar cell device. The short-circuit current density reached 28.31 mA/cm^2^, and the fill factor reached 70.25%. Subsequent to this research, further research is necessary to explore donor materials that enhance acceptor compatibility, facilitating the incorporation of a third component.

## Figures and Tables

**Table 1:** Key parameters of devices with different doping ratios of D18-Cl.

PM6:D18-Cl:Y6	*V_OC_* (V)	*J_SC_* (mA/cm^2^)	FF (%)	PCE (%)
1:0:1.2	0.82	25.36	64.62	13.52
0.9:0.1:1.2	0.82	25.95	67.73	14.55
0.8:0.2:1.2	0.81	28.13	70.25	16.08
0.7:0.3:1.2	0.80	28.01	69.55	15.62

## Data Availability

The raw data can be obtained from the corresponding authors upon reasonable request.

## Supplementary Materials

The following are available online at https://www.mdpi.com/article/10.3390/nano13212899/s1, Figure S1: Fill factors and other values for four doping ratios.

## References

[1] Hu Z., Wang J., Ma X., Gao J., Xu C., Yang K., Wang Z., Zhang J., Zhang F. (2020). A critical review on semitransparent organic solar cells. Nano Energy.

[2] Jia Z., Qin S., Meng L., Ma Q., Angunawela I., Zhang J., Li X., He Y., Lai W., Li N. (2021). High performance tandem organic solar cells via a strongly infrared-absorbing narrow bandgap acceptor. Nat. Commun..

[3] Jiang K., Wei Q., Lai J.Y., Peng Z., Kim H.K., Yuan J., Ye L., Ade H., Zou Y., Yan H. (2019). Alkyl Chain Tuning of Small Molecule Acceptors for Efficient Organic Solar Cells. Joule.

[4] Liao C.Y., Chen Y., Lee C.C., Wang G., Teng N.W., Lee C.H., Li W.L., Chen Y.K., Li C.H., Ho H.L. (2020). Processing Strategies for an Organic Photovoltaic Module with over 10% Efficiency. Joule.

[5] Dou L.T., You J.B., Yang J., Chen C.C., He Y.J., Murase S., Moriarty T., Emery K., Li G., Yang Y. (2012). Tandem polymer solar cells featuring a spectrally matched low-bandgap polymer. Nat. Photonics.

[6] Meng L.X., Zhang Y.M., Wan X.J., Li C.X., Zhang X., Wang Y.B., Ke X., Xiao Z., Ding L.M., Xia R.X. (2018). Organic and solution-processed tandem solar cells with 17.3% efficiency. Science.

[7] You J.B., Dou L.T., Yoshimura K., Kato T., Ohya K., Moriarty T., Emery K., Chen C.C., Gao J., Li G. (2013). A polymer tandem solar cell with 10.6% power conversion efficiency. Nat. Commun..

[8] Chen W.Q., Zhang Q.C. (2017). Recent progress in non-fullerene small molecule acceptors in organic solar cells (OSCs). J. Mater. Chem. C.

[9] Li C., Fu H.T., Xia T., Sun Y.M. (2019). Asymmetric Nonfullerene Small Molecule Acceptors for Organic Solar Cells. Adv. Energy Mater..

[10] Lin Y.Z., Ma L.C., Li Y.F., Liu Y.Q., Zhu D.B., Zhan X.W. (2014). Small-Molecule Solar Cells with Fill Factors up to 0.75 via a Layer-by-Layer Solution Process. Adv. Energy Mater..

[11] Zhang Z.Z., Yuan J., Wei Q.Y., Zou Y.P. (2018). Small-Molecule Electron Acceptors for Efficient Non-fullerene Organic Solar Cells. Front. Chem..

[12] Gao J.H., Ma X.L., Xu C.Y., Wang X.L., Son J.H., Jeong S.Y., Zhang Y., Zhang C.X., Wang K., Niu L.B. (2022). Over 17.7% efficiency ternary-blend organic solar cells with low energy-loss and good thickness-tolerance. Chem. Eng. J..

[13] Li M.Y., Pan Y.Q., Sun G.Y., Geng Y. (2021). Charge Transfer Mechanisms Regulated by the Third Component in Ternary Organic Solar Cells. J. Phys. Chem. Lett..

[14] Wang Z.Y., Zhang Y.J., Zhang J.Q., Wei Z.X., Ma W. (2016). Optimized “Alloy-Parallel” Morphology of Ternary Organic Solar Cells. Adv. Energy Mater..

[15] Zhang K.N., Yang X.Y., Niu M.S., Wen Z.C., Chen Z.H., Feng L., Feng X.J., Hao X.T. (2019). Modulating the morphology and molecular arrangement via the well-compatible polymer donor in multiple working mechanisms interwined ternary organic solar cells. Org. Electron..

[16] Cui Y., Yao H.F., Zhang J.Q., Zhang T., Wang Y.M., Hong L., Xian K.H., Xu B.W., Zhang S.Q., Peng J. (2019). Over 16% efficiency organic photovoltaic cells enabled by a chlorinated acceptor with increased open-circuit voltages. Nat. Commun..

[17] Gao W., An Q.S., Hao M.H., Sun R., Yuan J., Zhang F.J., Ma W., Min J., Yang C.L. (2020). Thick-Film Organic Solar Cells Achieving over 11% Efficiency and Nearly 70% Fill Factor at Thickness over 400 nm. Adv. Funct. Mater..

[18] Hadmojo W.T., Wibowo F.T.A., Lee W., Jang H.K., Kim Y., Sinaga S., Park M., Ju S.Y., Ryu D.Y., Jung I.H. (2019). Performance Optimization of Parallel-Like Ternary Organic Solar Cells through Simultaneous Improvement in Charge Generation and Transport. Adv. Funct. Mater..

[19] Liao S.H., Jhuo H.J., Cheng Y.S., Chen S.A. (2013). Fullerene Derivative-Doped Zinc Oxide Nanofilm as the Cathode of Inverted Polymer Solar Cells with Low-Bandgap Polymer (PTB7-Th) for High Performance. Adv. Mater..

[20] Lu L.Y., Xu T., Chen W., Landry E.S., Yui L.P. (2014). Ternary blend polymer solar cells with enhanced power conversion efficiency. Nat. Photonics.

[21] Ma X.L., An Q.S., Ibraikulov O.A., Lévêque P., Heiser T., Leclerc N., Zhang X.L., Zhang F.J. (2020). Efficient ternary organic photovoltaics with two polymer donors by minimizing energy loss. J. Mater. Chem. A.

[22] Yuan J., Zhang Y.Q., Zhou L.Y., Zhang G.C., Yip H.L., Lau T.K., Lu X.H., Zhu C., Peng H.J., Johnson P.A. (2019). Single-Junction Organic Solar Cell with over 15% Efficiency Using Fused-Ring Acceptor with Electron-Deficient Core. Joule.

[23] Campoy-Quiles M., Kanai Y., El-Basaty A., Sakai H., Murata H. (2009). Ternary mixing: A simple method to tailor the morphology of organic solar cells. Org. Electron..

[24] Lin Y.C., Su Y.W., Li J.X., Lin B.H., Chen C.H., Chen H.C., Wu K.H., Yang Y., Wei K.H. (2017). Energy transfer within small molecule/conjugated polymer blends enhances photovoltaic efficiency. J. Mater. Chem. A.

[25] Zhao W.C., Li S.S., Yao H.F., Zhang S.Q., Zhang Y., Yang B., Hou J.H. (2017). Molecular Optimization Enables over 13% Efficiency in Organic Solar Cells. J. Am. Chem. Soc..

[26] Cui Y., Yao H.F., Hong L., Zhang T., Xu Y., Xian K.H., Gao B.W., Qin J.Z., Zhang J.Q., Wei Z.X. (2019). Achieving Over 15% Efficiency in Organic Photovoltaic Cells via Copolymer Design. Adv. Mater..

[27] Lin Y.C., Chen C.H., Tsai B.S., Hsueh T.F., Tsao C.S., Tan S., Chang B., Chang Y.N., Chu T.Y., Tsai C.E. (2023). Alkoxy- and Alkyl-Side-Chain-Functionalized Terpolymer Acceptors for All-Polymer Photovoltaics Delivering High Open-Circuit Voltages and Efficiencies. Adv. Funct. Mater..

[28] Pan M.A., Lau T.K., Tang Y.B., Wu Y.C., Liu T., Li K., Chen M.C., Lu X.H., Ma W., Zhan C.L. (2019). 16.7%-efficiency ternary blended organic photovoltaic cells with PCBM as the acceptor additive to increase the open-circuit voltage and phase purity. J. Mater. Chem. A.

[29] Cai Y.H., Li Y., Wang R., Wu H.B., Chen Z.H., Zhang J., Ma Z.F., Hao X.T., Zhao Y., Zhang C.F. (2021). A Well-Mixed Phase Formed by Two Compatible Non-Fullerene Acceptors Enables Ternary Organic Solar Cells with Efficiency over 18.6%. Adv. Mater..

[30] Gurney R.S., Lidzey D.G., Wang T. (2019). A review of non-fullerene polymer solar cells: From device physics to morphology control. Rep. Prog. Phys..

[31] Li K., Wu Y.S., Tang Y.B., Pan M.A., Ma W., Fu H.B., Zhan C.L., Yao J.N. (2019). Ternary Blended Fullerene-Free Polymer Solar Cells with 16.5% Efficiency Enabled with a Higher-LUMO-Level Acceptor to Improve Film Morphology. Adv. Energy Mater..

[32] Lv R.Z., Chen D., Liao X.F., Chen L., Chen Y.W. (2019). A Terminally Tetrafluorinated Nonfullerene Acceptor for Well-Performing Alloy Ternary Solar Cells. Adv. Funct. Mater..

[33] Yu R.N., Yao H.F., Hou J.H. (2018). Recent Progress in Ternary Organic Solar Cells Based on Nonfullerene Acceptors. Adv. Energy Mater..

[34] Zhang Y., Liu D.L., Lau T.K., Zhan L.L., Shen D., Fong P.W.K., Yan C.Q., Zhang S.Q., Lu X.H., Lee C.S. (2020). A Novel Wide-Bandgap Polymer with Deep Ionization Potential Enables Exceeding 16% Efficiency in Ternary Nonfullerene Polymer Solar Cells. Adv. Funct. Mater..

[35] Zhou D., You W., Xu H.T., Tong Y.F., Hu B., Xie Y., Chen L. (2020). Recent progress in ternary organic solar cells based on solution-processed non-fullerene acceptors. J. Mater. Chem. A.

[36] Cai G.L., Li Y.H., Zhou J.D., Xue P.Y., Liu K., Wang J.Y., Xie Z.Q., Li G., Zhan X.W., Lu X.H. (2020). Enhancing Open-Circuit Voltage of High-Efficiency Nonfullerene Ternary Solar Cells with a Star-Shaped Acceptor. Acs Appl. Mater. Interfaces.

[37] Chang L.C., Sheng M., Duan L.P., Uddin A. (2021). Ternary organic solar cells based on non-fullerene acceptors: A review. Org. Electron..

[38] He D., Zhao F.W., Wang C.R., Lin Y.Z. (2022). Non-Radiative Recombination Energy Losses in Non-Fullerene Organic Solar Cells. Adv. Funct. Mater..

[39] Karki A., Vollbrecht J., Dixon A.L., Schopp N., Schrock M., Reddy G.N.M., Nguyen T.Q. (2019). Understanding the High Performance of over 15% Efficiency in Single-Junction Bulk Heterojunction Organic Solar Cells. Adv. Mater..

[40] Wang R., Zhang C.F., Li Q., Zhang Z.G., Wang X.Y., Xiao M. (2020). Charge Separation from an Intra-Moiety Intermediate State in the High-Performance PM6:Y6 Organic Photovoltaic Blend. J. Am. Chem. Soc..

[41] Zhang M., Zhu L., Zhou G.Q., Hao T.Y., Qiu C.Q., Zhao Z., Hu Q., Larson B.W., Zhu H.M., Ma Z.F. (2021). Single-layered organic photovoltaics with double cascading charge transport pathways: 18% efficiencies. Nat. Commun..

[42] Gao J.H., Gao W., Ma X.L., Hu Z.H., Xu C.Y., Wang X.L., An Q.S., Yang C.L., Zhang X.L., Zhang F.J. (2020). Over 14.5% efficiency and 71.6% fill factor of ternary organic solar cells with 300 nm thick active layers. Energy Environ. Sci..

