# Supplementary figures and images for: Improved Short-Circuit Current and Fill Factor in PM6:Y6 Organic Solar Cells through D18-Cl Doping
Source: Nanomaterials (Basel). 2023 Nov 3;13(21):2899. doi: 10.3390/nano13212899 (PMC10650114; doi:10.3390/nano13212899)

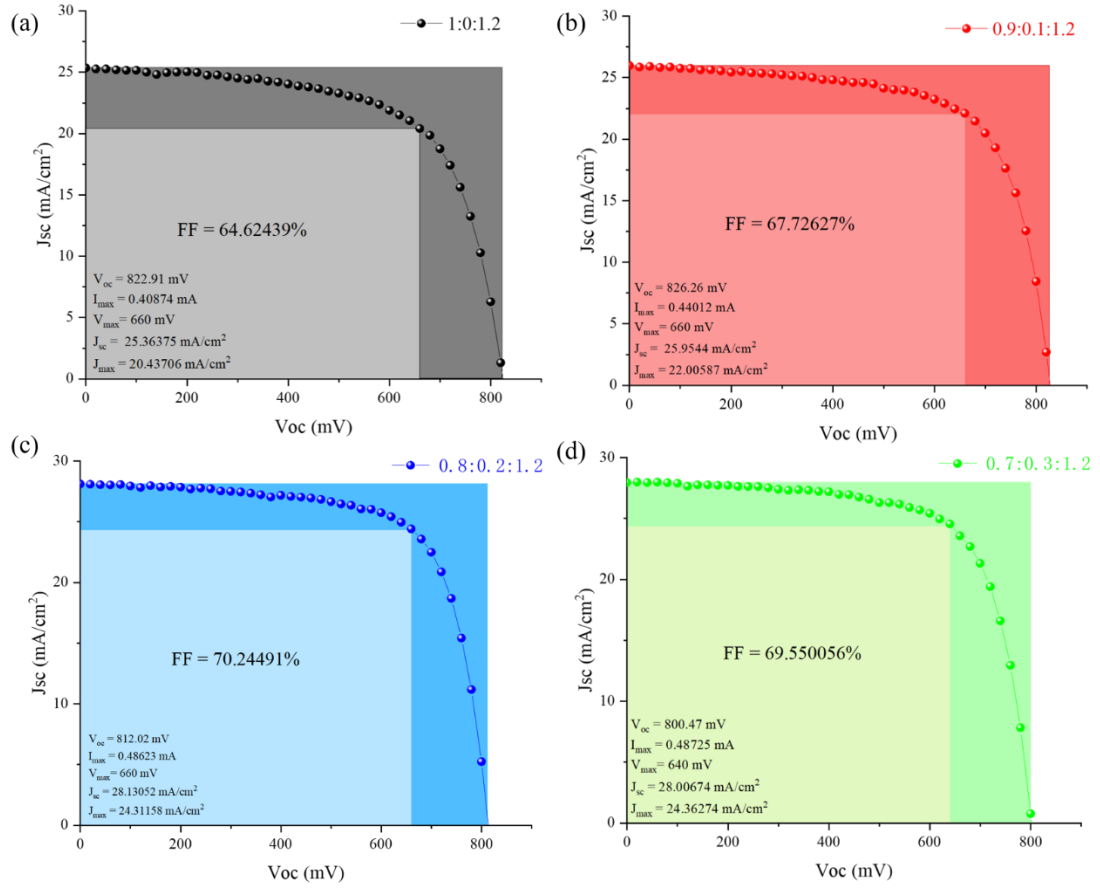

**Figure S1.** Filling factors and other values for four doping ratios. (a) 1:0:1.2; (b) 0.9:0.1:1.2; (c) 0.8:0.2:1.2; (d) 0.7:0.3:1.2.

Supplement: Supplementary file 1 [file nanomaterials-13-02899-s001.zip › nanomaterials-2674473-supplementary.pdf]
